# Anti-Tumor and Immune Enhancing Activities of Rice Bran Gramisterol on Acute Myelogenous Leukemia

**DOI:** 10.1371/journal.pone.0146869

**Published:** 2016-01-11

**Authors:** Somsuda Somintara, Vijittra Leardkamolkarn, Panawan Suttiarporn, Sugunya Mahatheeranont

**Affiliations:** 1 Department of Anatomy, Faculty of Science, Mahidol University, Bangkok, Thailand; 2 Department of Chemistry, Faculty of Science, Chiang Mai University, Chiang Mai, Thailand; Emory University, UNITED STATES

## Abstract

**Background:**

Acute myelogenous leukemia (AML) is a cancer of the blood that most commonly affects human adults. The specific cause of AML is unclear, but it induces abnormality of white blood cells that grow rapidly and accumulate in bone marrow interfering with the production and functions of the normal blood cells. AML patients face poor prognosis and low quality of life during chemotherapy or transplantation of hematopoietic stem cells due to the progressive impairment of their immune system. The goal of this study is to find natural products that have the potential to delay growth or eliminate the abnormal leukemic cells but cause less harmful effect to the body’s immune system.

**Methods and Findings:**

The unsaponified fraction of *Riceberry* rice bran (RBDS) and the main pure compound, gramisterol, were studied for cytotoxicity and biological activities in WEHI-3 cells and in the leukemic mouse model induced by transplantation of WEHI-3 cells intraperitoneally. In the *in vitro* assay, RBDS and gramisterol exerted sub-G1 phase cell cycle arrest with a potent induction of apoptosis. Both of them effectively decreased cell cycle controlling proteins (cyclin D1 and cyclin E), suppressed cellular DNA synthesis and mitotic division, and reduced anti-apoptosis Bcl-2 protein, but increased apoptotic proteins (p53 and Bax) and activated caspase-3 enzyme in the intrinsic cell death stimulation pathway. In leukemic mice, daily feeding of RBDS significantly increased the amount of immune function-related cells including CD3^+^, CD19^+^, and CD11b^+^, and elevated the serum levels of IFN-γ, TNF-α, IL-2, and IL-12β cytokines, but suppressed IL-10 level. At the tumor sites, CD11b^+^ cells were polarized and became active phagocytotic cells. Treatment of mice normal immune cells with gramisterol alone or a combination of gramisterol with cytokines released from RBDS-treated leukemic mice splenocytes culture synergistically increased pSTAT1 transcriptional factor that up-regulated the genes controlling cell survival and function. Phosphorylation of STAT1 was absent in WEHI-3. Instead, similar treatments significantly decreased pSTAT3 signaling that regulates transcription of genes controlling tumor growth and proliferation.

**Conclusions:**

Rice bran gramisterol possesses a promising anti-cancer effect against a tumor of white blood cells and induces the production of anti-cancer immune-related cytokines. Gramisterol induces cell cycle arrest and apoptosis via suppression of pSTAT3 signaling control of tumor cells’ growth and progression. Gramisterol increased IFN-γ production and prevented the dysfunctional immune system of leukemic mice by enhancing pSTAT1 transcription signal controlling proliferation and functions of hematopoietic cells in the spleen. Together with IFN-γ, gramisterol efficiently facilitates leukemic mice immune system modulation leading to improvement of the AML condition. Administration of RBDS containing gramisterol potentiates immune recovery of leukemic mice and extends their survival. This finding encourages the medicinal application of rice bran gramisterol as a palliative treatment or an alternative agent for future drug development against AML.

## Introduction

Acute myelogenous leukemia (AML) is a type of blood cancer that most commonly affects adult humans and leads to death. Although the incidence rate today is considered low, it is continuously rising because of the increasing aged population that is more exposed to several risk factors including repetitive exposure to chemicals [1–3], ionizing radiation [4–6], and chemotherapies [7, 8]. The affected persons have impaired immune function. The clinical signs and symptoms of AML are varied individually including weight loss, weakness, abnormal blood cells, infection, and enlargement of liver and spleen.The first line of AML treatment is induction of chemotherapy, most commonly by continuous infusion of cytarabine and an anthracycline [9, 10]. For patients at high risk of relapse, hematopoietic stem cell transplantation is usually applied [11]. Such a chemotherapeutic regimen has potential to induce progressive impairment of the immune system therefore, not all patients are able to tolerate the aggressive therapies. Recently, research investigators are searching for new anti-leukemic drugs and/or appropriate chemo-adjuvants to relieve the side effects of the existing drugs.

Rice bran contains significant amounts of phytochemicals including phytosterols that are beneficial to human health. Among them, γ-oryzanol, tocopherols, and tocotrienols constituents in the bran extract of pigmented rice are well accepted for their antioxidant activities [12]. They are most recognized for efficacy in alleviation of illness conditions including diabetes, kidney stones, fatty liver, cancer and cardiovascular diseases [13, 14]. Significant amounts of linoleic acid, phenols, anthocyanins, β-sitosterol, campesterol, stigmasterol, 22,23-dihydrostigmasterol, ergost-4,6,22-trien-3-ol and a cycloartane-type triterpene are also found in the bran extract of rice [15, 16], especially in the Thai black rice cultivar, *Riceberry* [17]. A mixture of these constituents or the crude extract of *Riceberry* bran have shown health benefits in diabetic rats [18], and its dichloromethane (DCM) extract has demonstrated anti-cancer effect against several cancer cell lines with various degree of effectiveness [17]. Our research group is especially interested in the bioactive compounds that potentiate such diversities. We have successively sub-fractioned the unsaponified DCM extract of the rice bran by employing HPLC purification technique and isolated the individual compounds which were mainly classified in the phytosterols group [19]. In the present study, the major phytosterol, gramisterol, was evaluated for anti-tumor activity and the mode of the action in a mice leukemic cell line and mice normal immune cells. The compound’s effectiveness was also investigated in a leukemic mouse model. The findings suggest that gramisterol in the unsaponified DCM fraction of *Riceberry* bran (RBDS) effectively improves the immune system of the leukemic mice and it has potential for future anti-cancer drug development against human leukemia.

## Methods

### Cell Line and Culture Conditions

Murine myelomonocytic leukemia cell line (WEHI-3) was purchased from the American Type Culture Collection (Rockville, MD, USA). The cells were propagated in 75 cm^2^ cell culture flasks and grown in RPMI 1640 medium supplemented with 100 Units/mL penicillin and 100 μg/mL streptomycin, 1% glutamine and 10% fetal bovine serum (Thermo Fisher Scientific, Waltham, MA, USA) at 37°C under a humidified 5% CO_2_ atmosphere.

### Rice Bran Extract and Pure Compound Preparation

A black non-glutinous rice, cv. *Riceberry*, used in this study, was grown at the experimental rice field of Kasetsart University, NakornPathom province, Thailand. The bran was successively extracted by hexane and dichloromethane, followed by saponification with 0.5 M ethanolic KOH solution to obtain the RBDS. The RBDS was further sub-fractioned using an HPLC purification system until the main phytosterols were obtained and characterized as detailed in our previous report [19]. The major compound identified from this method was gramisterol. The RBDS and gramisterol were prepared in stock (in absolute ethanol) and diluted fresh with the culture media for use in each experiment.

### Cytotoxicity (MTT) Assay

To determine their cytotoxicity, RBDS at concentrations of 0, 25, 50, 100, 200 and 300 μg/mL was added to 2x10^5^ WEHI-3 cells grown for 24 h in 96-well-plates in a humidified 5% CO_2_ incubator at 37°C. The treated cells were incubated for 24, 48 and 72 h. Gramisterol at concentrations of 0, 0.25, 0.5, 1, 2, 4 and 8 μM was incubated with WEHI-3 cells for 24 h. After incubation, the culture supernatant was replaced with 10 μL of MTT solution (Sigma Aldrich, St.Louis, MO, USA) and incubated at 37°C for 4 h. The color products were measured by spectrophotometer at 570 nm and 690 nm. The experiments were performed in triplicate.

### Cell Cycle Analysis

WEHI-3 cells (1x10^6^ cells) were treated with RBDS (0, 50 and 100 μg/mL) or gramisterol (0, 1 and 2 μM) for 24 h. The treated cells were pelleted, centrifuged, washed twice with cold PBS, and fixed with 70% ethanol at -20°C for 24h. Then, the cells were stained with a nuclear staining dye containing 50 μg/mL propidium iodide (Merck, Frankfurt, Germany), 50 μg/mL RNase A, and 3.8 mM/L sodium citrate (Sigma Aldrich, St. Louis, MO, USA) at 37°C for 30 min. The DNA contents at different phases of the cell cycle were measured by flow cytometry using a FACScanto apparatus (Becton Dickinson, San Diego, CA, USA) and analyzed with the FACSDiva version 6.1.1 software (Becton Dickinson, San Jose, CA, USA).

### Apoptosis Determination

For apoptosis analysis, RBDS-treated WEHI-3 cells (5x10^4^ cells) were evaluated for apoptotic phenomena after 24 and 48 h incubation. The treated cells were cold fixed with methanol, air dried and stained with DAPI nuclear staining dye (Boehringer, Ingelheim, Germany) at 37°C for 30 min. Apoptosis hallmarks such as cell shrinkage, membrane bleb, and chromatin condensation were examined under a fluorescence microscope. In addition, DNA fragmentation in the treated cells was analyzed by DNA isolation using TRI REAGENT (Molecular Research Center, Inc., Cincinnati, OH, USA) and processed in 1.5% agarose gel electrophoresis. The gel was stained with ethidium bromide and visualized under an ultraviolet transilluminator (UVP BioImaging System, USA). Stages of cells under apoptosis were analyzed by annexin-V staining, using FITC Annexin-V Apoptosis Detection Kit I (BD Pharmingen, San Diego, CA, USA), accordingly to the manufacturer’s instruction. Briefly, some treated cells (1x10^6^ cells/mL) were pelleted and suspended in 1X binding buffer and resuspended in 100 μL buffer. The cell suspension was transferred to a flow cytometry tube. 5 μL of FITC Annexin-V plus 5 μL of PI were added, gently mixed and incubated at room temperature for 15 min in the dark. 400 μL of 1X binding buffer was added to each tube before flow cytometry analysis (FACSCanto, Becton Dickinson, San Diego, CA, USA).

### Western Blotting

Proteins involved in cell cycle and apoptosis control in the treated cells were measured by Western blot analysis. WEHI-3 cells were treated with RBDS at a concentration of 0, 50, 100, 200 μg/mL and gramisterol at doses of 0, 1, 2 and 3 μM for 24 h. Then the cells were harvested, washed, and centrifuged. The cell pellets were lysed with RIPA buffer (25 mM Tris-HCl pH 7.6, 150 mM NaCl, 1% NP-40, 1% sodium deoxycholate, 0.1% SDS) containing protease inhibitor cocktail (Merck, Frankfurt, Germany). 30 μg of cellular proteins from each treatment was separated by 12% SDS polyacrylamide gel, and transferred to Amersham Hybond ECL nitrocellulose membrane (GE Healthcare, Piscataway, NJ, USA). The membrane was blocked with 3% BSA in TBST (100 mM Tris-base, 150 mM NaCl, 0.1% Tween20) for 2 h, and incubated with 1:1000 dilution of rabbit anti-mouse monoclonal antibodies against cyclin E, cyclin D1, Bcl-2, Bax, p53, caspase-3, cleaved caspase-3, and β-actin (Cell Signaling Technology, Beverly, MA, USA) overnight at 4°C. The membrane was washed and incubated with 1:2000 dilution of goat anti-rabbit IgG (H&L)-horseradish peroxidase (HRP) conjugated antibody (Cell Signaling Technology, Beverly, MA, USA) for 2 h at room temperature. The protein bands on the membrane were stained with ECL Prime Western Blotting Detection Reagent (GE Healthcare, Buckinghamshire, United Kingdom) and exposed to X-ray film under dark room conditions. The films were photographed and analyzed using ImageJ software (http://rsbweb.nih.gov/ij/download.html).

### Animal Experiment

60 BALB/c mice (weighing 22–28 g) were obtained from the National Laboratory Animal Center of Mahidol University, Thailand. All mice were maintained at 22±2°C on a 12 h dark/light cycle and provided with the standard pellet diet including drinking water *ad libitum*. The mice were acclimated for a week before the experiment. They were randomly divided into 6 subgroups (10 mice each): Group 1, normal control (distilled water), Group 2, vehicle control (olive oil), Group 3, leukemic control (WEHI-3), Group 4, leukemia + vehicle (WEHI-3 + olive oil), Group 5, leukemia + RBDS 3 mg/kg (WEHI-3 +RBDS 3 mg/kg), and Group 6, leukemia + RBDS 9 mg/kg (WEHI-3 + RBDS 9 mg/kg). Leukemia was induced in mice by an intraperitoneal (i.p) injection of WEHI-3 cells (1 x 10^5^ cells in 100 μL PBS) [20]. Except the leukemic control group, the leukemic mice were pretreated by gavage tubing with the olive oil or RBDS daily for 1 wk before leukemia induction, then, they were continuously fed with RBDS or olive oil daily. Mice body weight was recorded daily for 28 d.

At the end of the experiment, the mice were weighed, bled from submandibular vessels and euthanized with CO_2_.

#### Organ Weights and Histopathological Assessment

At the end of the experiment, mice spleens and livers were harvested and weighed separately. The organ index was calculated by dividing the organ weight by the mice body weight. The organs were cut into small pieces, fixed in 4% formaldehyde and embedded in paraffin. Tissues were sectioned (5 μm) and stained with Hematoxylin & Eosin, then examined under the light microscope.

#### Analysis of Peripheral Blood Immune Cells

White blood cells were isolated by lysis of red blood cells using FACS lysing solution according to the manufacturer’s instruction (BD Biosciences, San Jose, CA, USA). The pellet was fixed with 1% formaldehyde and examined for the presentation of the immune cells by staining with specific cell surface markers using the following conjugated antibodies: anti-CD3-FITC (for T cells), CD19-PE (for B cells), CD11b-FITC (for monocytes, neutrophil etc.) and Mac-3-PE (for macrophages) (Santa Cruz Biotechnology, Inc., CA, USA). The surface markers’ intensity and level were measured and analyzed by flow cytometry (FACSCanto, Becton Dickinson, San Diego, CA, USA).

#### Measurement of Serum Cytokine Level

Serum cytokines involved in the immune response, interferon gamma (IFN-γ), interleukin-12β (IL-12β), tumor necrosis factor-alpha (TNF-α), interleukin-2 (IL-2) and interleukin-10 (IL-10), were measured individually from each mouse (four mice per group) by enzyme-linked immunosorbent assay (ELISA) using anti-mouse IFN-γ, IL-12β, TNF-α, IL-2, and IL-10 (eBioscience, Inc., San Diego, CA, USA) following the manufacturer’s instruction.

#### Phagocytic Activity of Peritoneal Macrophages

Macrophages were isolated from mice peritoneal lavage and their functional ability was determined using the pHrodo^TM^ Red Dextran beads assay (Molecular Probes, Carlsbad, CA, USA). Briefly, the isolated macrophages (5x10^5^ cells) were grown in 6-well plates in RPMI 1640 medium containing standard constituents and conditions for 24 h. Then, they were placed in a microtube, washed with PBS at pH 7.4 and centrifuged at 12,000 g, 4°C for 10 min. The cells were resuspended in pHrodo^TM^ Red Dextran beads to the final concentration 50 μg/mL and incubated at 37°C for 30 min in dark. The stained cells were washed with PBS, immediately analyzed by flow cytometry (FACSCanto, Becton Dickinson, USA) and observed under the fluorescence microscope.

#### Signaling Activity of Gramisterol on Normal Immune Cells and Tumor Cells

Splenocytes and peritoneal cells were isolated from spleen and peritoneal lavage of the normal BALB/c mice. The primary cells were cultured in RPMI 1640 medium containing 1% penicillin-streptomycin (100 U/mL penicillin and 100 μg/mL streptomycin), 1% glutamine and 10% fetal bovine serum, at 37°C under a humidified 5% CO_2_ for 24 h before treatments with (i) gramisterol (2 μM), (ii) culture supernatant containing cytokines released from RBDS-treated leukemic mice spleen cells (supplemented with 100 ng/mL IFN-γ) (R&D Systems, Inc., Minneapolis, MN, USA), and (iii) combined gramisterol (2 μM) + serum containing cytokines from RBDS treated mice (supplemented with 100 ng/mL IFN-γ). The biological activity and the mechanism by which gramisterol exerted activity on these immune cells’ function and survival were examined by Western blot analysis. Briefly, the whole cell lysates were run on an 8% SDS-PAGE gel and immunoblotted with 1:1000 dilution of rabbit antibodies against mouse phospho-STAT1 (Try701), phospho-STAT3 (Try705) and β-actin (Cell Signaling Technology, Beverly, MA, USA) for 24 h. After washing off the excess primary antibodies, the membranes were incubated with 1:2000 dilution of goat anti-rabbit IgG (H&L)-HRP conjugated antibody (Cell Signaling Technology, Beverly, MA, USA). The activity of gramisterol treatment on WEHI-3 tumor cells was determined in parallel for comparison.

### Statistical Analysis

The data were analyzed using one-way ANOVA, followed by Tukey post hoc test. All data were expressed as mean±SEM and the significant difference between groups was considered at *p* < 0.05.

### Ethics Statement

The animal experiments were carried out in strict accordance with the recommendations in the Guide for the Care and Use of Laboratory Animals of the National Institutes of Health. This animal ethic protocol was approved by the Faculty of Science, Mahidol University Animal Care and Use Committee SCMU-ACUC Review (No. MUSC54-018-228/1). One week after leukemia induction, mice were monitored for signs of sickness, pain, distress, or suffering three times/day. Body weight was recorded daily. Moribund conditions from systemic organ failure were expected since leukemic cells can travel along the blood vessel and spread to many vital organs including liver, spleen, kidney, and lung. Once clinical indicators of humane serious illness such as dyspnea (difficulty breathing), tachypnea (rapid breathing), weakness, ataxia (lack of coordination), hypoactivity, stupor, coma, or weight loss was found, the mice were considered at endpoint and euthanized. The CO_2_ euthanasia was performed, in complying with the Guide for Care and Use of Laboratory Animals of the National Institutes of Health, at less than 5 psi (pounds per square inch), approximately 2 liters/minute flow rate, for 3–5 minutes or until the animals were completely dead.

## Results

### Effects of RBDS and Gramisterol on WEHI-3 Cells

**Fig 1:**
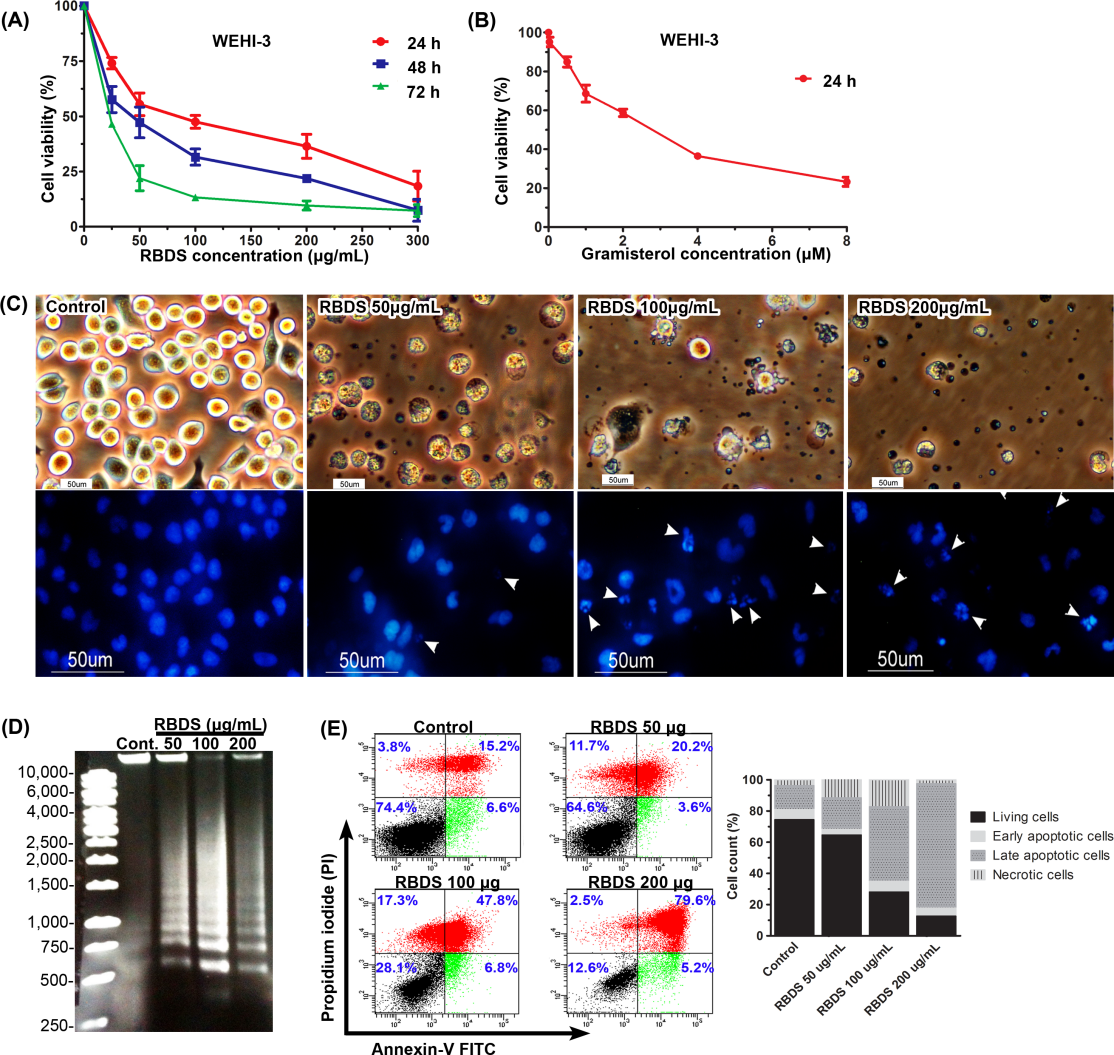
Effects of RBDS and gramisterol on cell viability and apoptosis. (A), (B) MTT assay of WEHI-3 cells treated with different concentrations of RBDS for 24, 48 and 72 h, and gramisterol for 24 h. Percent cell viability was calculated relative to the untreated control. The experiments were performed in triplicate and data are presented as mean±SEM. Significant difference was considered at *p*<0.05 compared with the control. (C) Phase contrast photographs and DAPI-DNA staining of WEHI-3 cells after treatment with RBDS 0, 50, 100 and 200 μg/mL for 48 h. The apoptotic bodies are indicated by white arrowheads. (D) Gel electrophoresis of DNA extracted from RBDS treated-WEHI-3 for 48 h. DNA ladder or fragmentation indicated apoptosis event in the cells. (E) Flow cytometry analysis of WEHI-3 cells after 48 h RBDS-treatment and annexin-V staining. Percent cell count of apoptosis detection was demonstrated from the average of two experiments.

RBDS reduced WEHI-3 cells viability in a concentration- and time- dependent manner. The 50% inhibition of cell growth (IC_50_) by RBDS at 24, 48 and 72h was achieved by 51.83±0.07, 34.64±0.06, and 14.66±0.07 μg/mL, respectively (Fig 1A). The pure compound, gramisterol, also reduced WEHI-3 cells viability in a concentration-dependent manner. The IC_50_ by gramisterol at 24 h was 1.53±0.06 μM (Fig 1B). RBDS-treated cells manifested loss of membrane integrity, condensation of nuclei and subsequent induction of apoptotic body formation (Fig 1C) and DNA fragmentation (Fig 1D). Apoptosis detection was verified in WEHI-3 cells by Annexin-V/flow cytometry analysis. At 48 h post-treatment, an increased concentration of RBDS increased percent of late apoptosis cells (Fig 1E).

**Fig 2:**
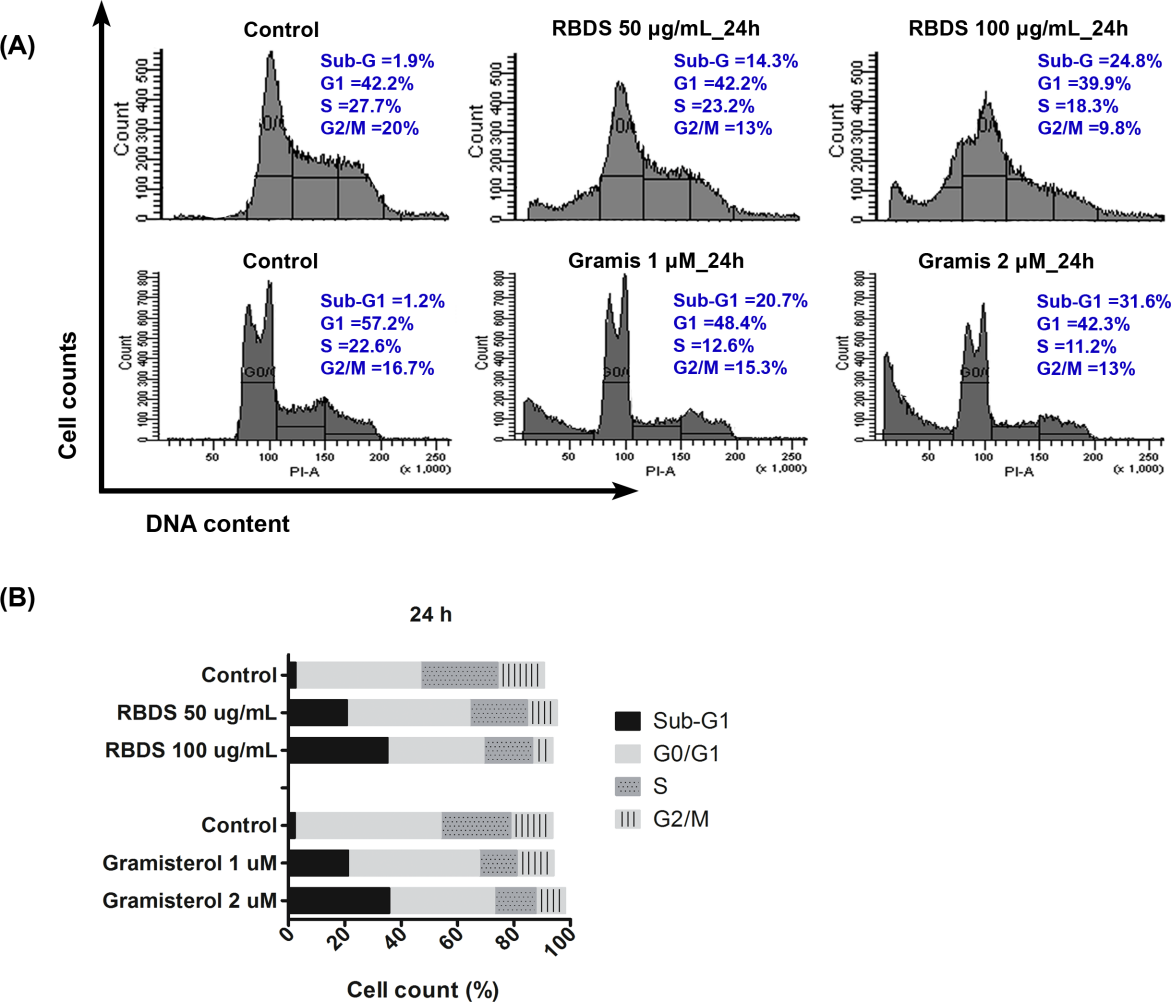
Effects of RBDS and gramisterol on the cell cycle. (A) Representative flow cytometry analysis of DNA contents (PI staining) in WEHI-3 cells after 24 h treatment with RBDS (0, 50 and 100 μg/mL) and gramisterol (0, 1 and 2 μM). (B) Bar graphs and histograms show DNA distribution in each phase of cell cycle. The experiments were performed in duplicate and data are presented as average.

RBDS treatment (50 and 100 μg/mL, for 24 h and 48 h) and gramisterol treatment (1 and 2 μM, for 24 h) induced a similar pattern of cell cycle arrest in WEHI-3 cells. Flow cytometry analysis revealed an increase of DNA contents in the sub-G1 phase and a slight decrease of the DNA contents in the subsequent phases in a concentration dependent effect (Fig 2A) reflecting the inhibition of the cell cycle. The average percentage of cells counted at each phase of the cell cycle from duplicate experiments of RBDS and gramisterol after 24 h treatment is shown in Fig 2B.

### Western Blot Analysis of Proteins Involved in Cell Cycle Control and Apoptosis

**Fig 3:**
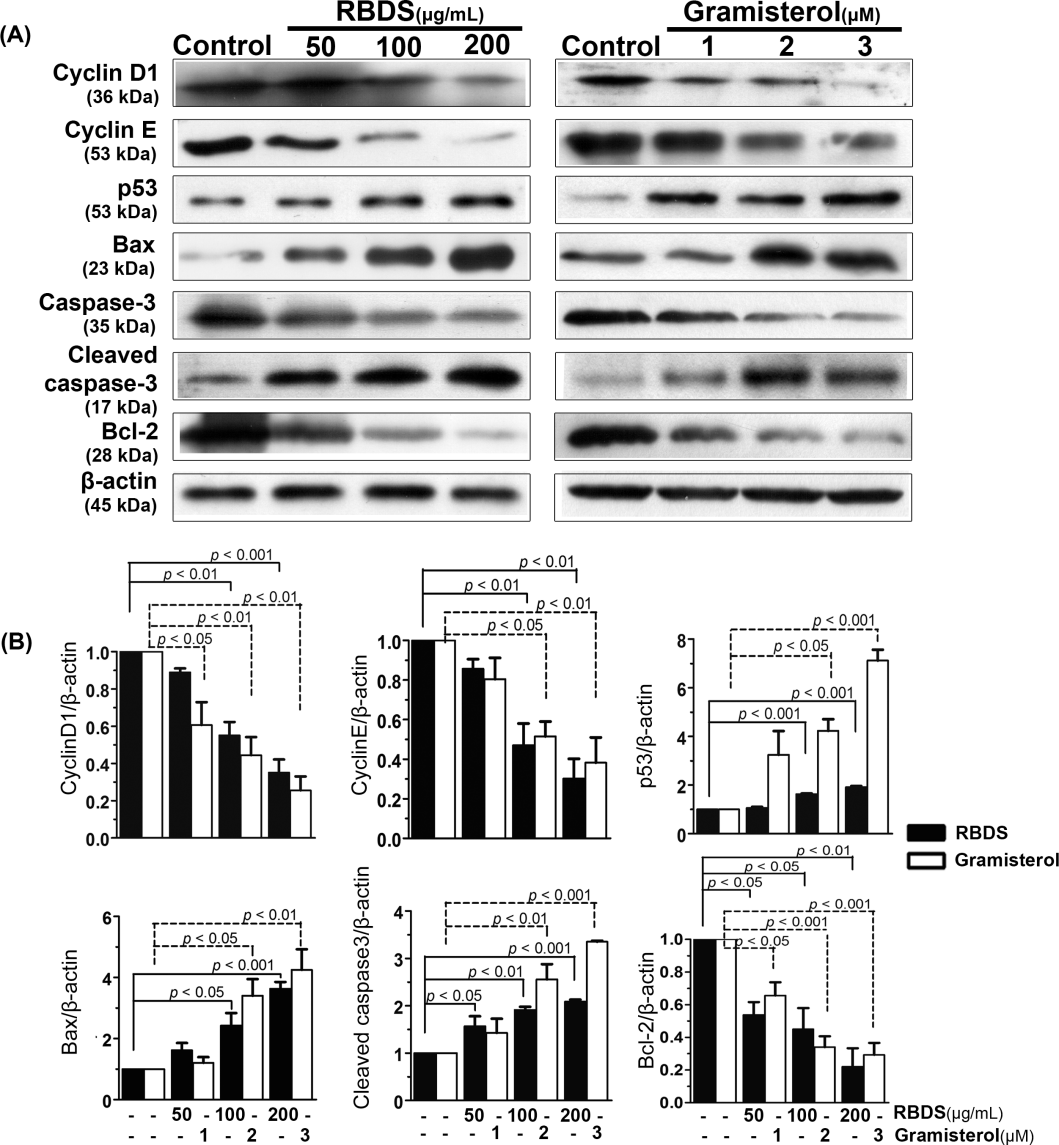
Effects of RBDS and gramisterol on cell cycle and apoptosis proteins. (A) Representative immunoblotting assay of WEHI-3 cells treated with RBDS (0, 50, 100 and 200 μg/mL) and gramisterol (0, 1, 2 and 3 μM) for 24 h. The experiments were performed in triplicate. (B) The protein bands density were measured semi-quantitatively and analyzed using ImageJ software. Data are expressed as mean±SEM (n = 3).

The Western blot analysis indicated that RBDS and gramisterol decreased the level of cell cycle proteins cyclin E and cyclin D1, and anti-apoptotic Bcl-2 protein, but increased the tumor suppressor p53, pro-apoptosis Bax proteins, and cleaved caspase-3 enzyme in WEHI-3 cells in a concentration dependent response (Fig 3A). The proteins content relative to the internal control (β-actin) from the RBDS and gramisterol treatment were compared to the untreated (culture media treatment) control (Fig 3B).

### Effects of RBDS Containing Gramisterol on Mice Leukemic Model

#### Body Weight and Percent Survival of Mice

**Fig 4:**
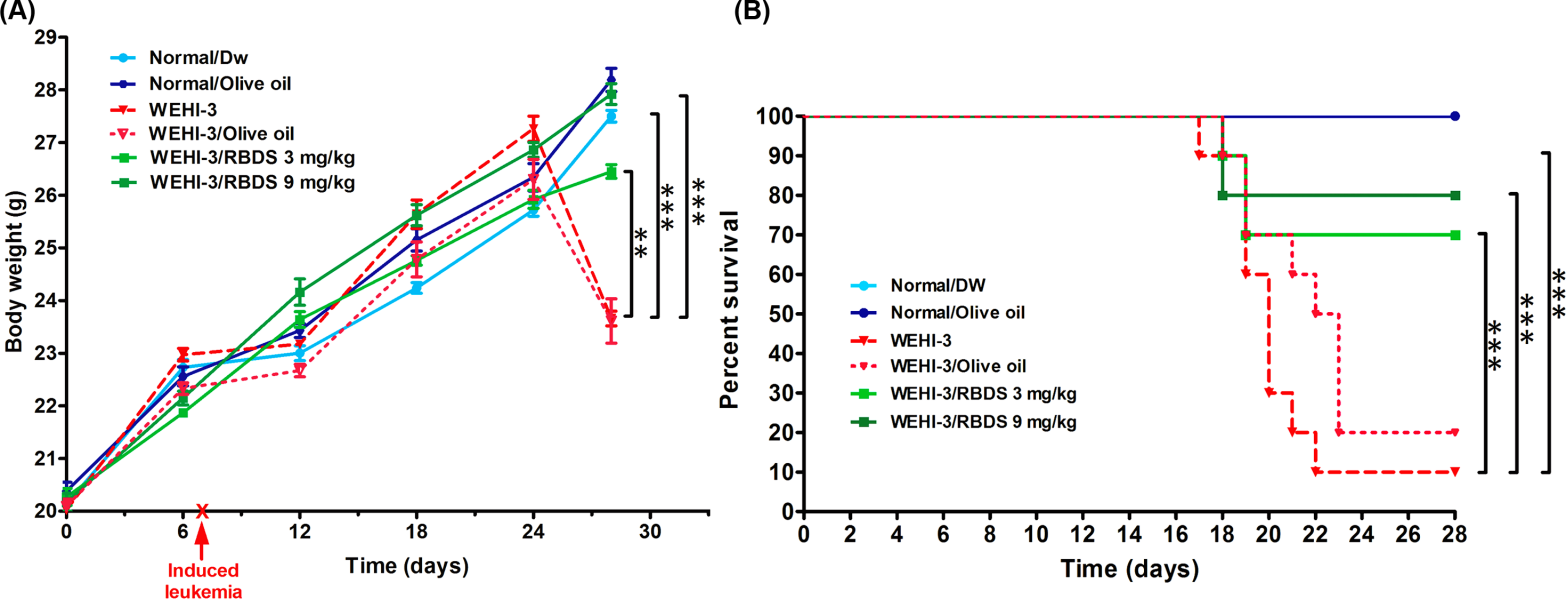
Effects of RBDS treatment on the body weight and percent survival. (A) Body weight of leukemic mice treated with RBDS (WEHI-3/RBDS 3 mg/kg and WEHI-3/RBDS 9 mg/kg) compared with normal mice and untreated-leukemic mice (WEHI-3). (B) Percent survival of WEHI-3/RBDS 3 mg/kg and WEHI-3/RBDS 9 mg/kg mice were significantly higher than the untreated-WEHI-3. Data are presented as mean±SEM (n = 10). **p< 0.01, ***p< 0.001 were considered significant differences compared to WEHI-3.

The average body weight of leukemia-bearing mice in the untreated group (WEHI-3) and the olive oil-treated WEHI-3 group gradually increased from the beginning then rapidly declined after 24 d. The body weights of the RBDS-treated (WEHI-3/RBDS 3 mg/kg and WEHI-3/RBDS 9 mg/kg) increased at approximately the same level as the normal control (Fig 4A). Toward the end of the experiment, some treated and untreated leukemic mice died, thus, mice were observed closely to collect data records, blood and organs. The percent survival of each group was calculated and is presented in Fig 4B. The RBDS-treated leukemic mice (WEHI-3/RBDS3 mg/kg and WEHI-3/RBDS 9 mg/kg) exhibited a concentration-related higher survival rate compared to the untreated-leukemic mice (Fig 4B).

#### Pathological Changes in Mice Spleen and Liver Tissues

**Fig 5:**
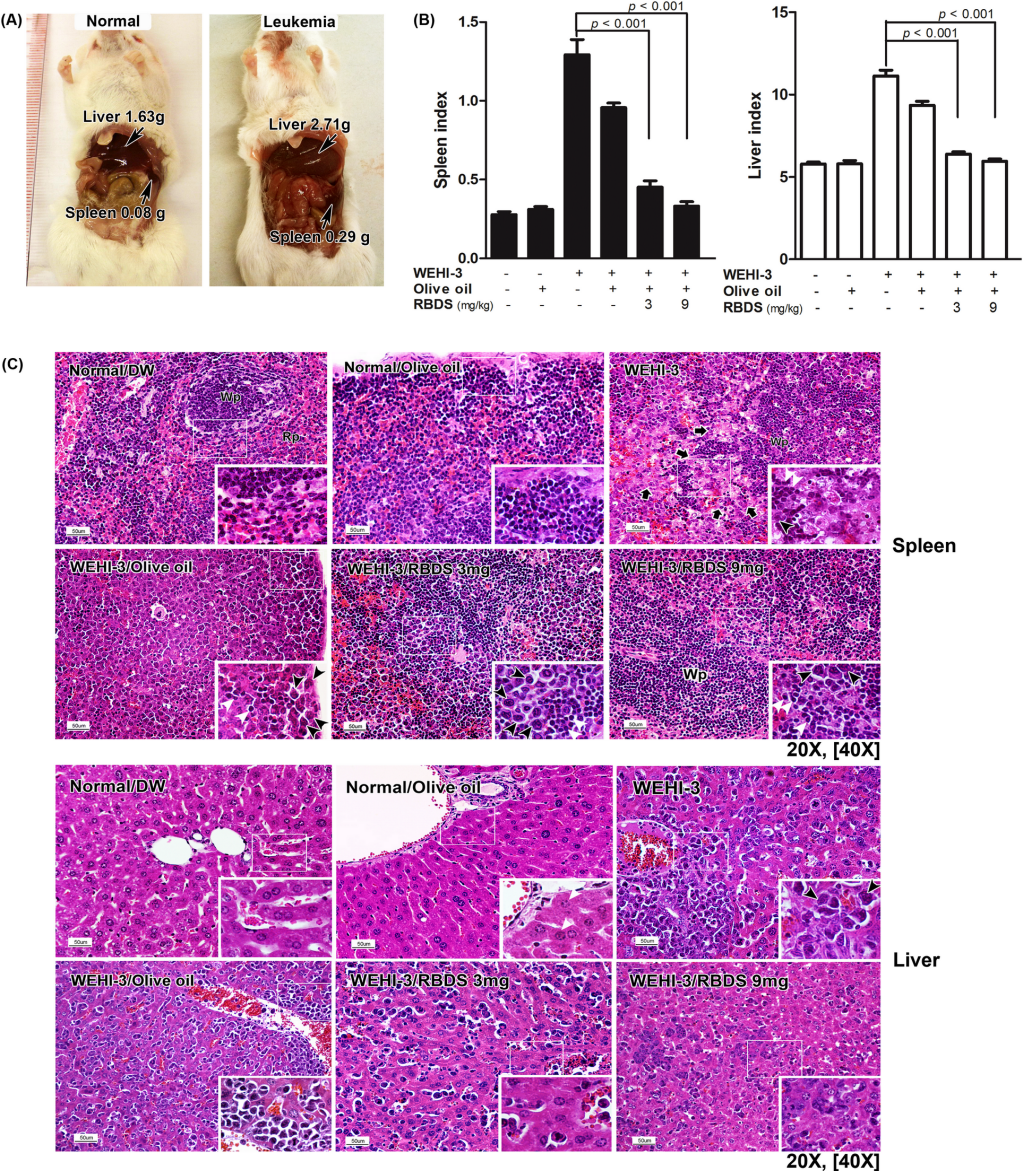
Pathological changes in spleen and liver. (A) Representative photograph of mice spleen and liver *in situ*. (B) Organ index was calculated from organ weight (g)/body weight (g) × 100. Data are expressed as mean±SEM. (n = 10). (C) Histopathological features of mice spleen and liver stained with Hematoxylin & Eosin (original magnification 20X; insets 40X). In spleen section: black arrows indicate invasion of leukemic cells into white pulps forming tumor necrotic foci. Black and white arrowheads indicate leukemic cells and lymphocytes, respectively. Abbreviations: Wp = white pulp, Rp = red pulp, C = capsule. In liver section: black and white arrowheads indicate leukemic cells and hepatocytes, respectively.

Leukemia-induced mice spleen and liver weighed at autopsy showed splenomegaly and hepatomegaly (Fig 5A). The RBDS treatment significantly reduced the abnormal organ size compared to the untreated-WEHI-3 and the olive oil treated controls (Fig 5B). Histopathological examination of the WEHI-3 induced leukemia tissues demonstrated a massive infiltration of leukemic cells in the mice spleen and liver tissues (Fig 5C). In the spleen, leukemic cells (nuclear and cellular pleomorphism) were present in the red pulp area diminishing the normal distribution of blood cells including RBC, megakaryocytes, reticular cells, monocytes, macrophages, neutrophils and hematopoietic foci. Also, the tumor cells replaced splenic parenchyma in the subcapsular and white pulp areas. In the liver, the normal hepatic cords architecture and hepatic cells were distorted. These observations were ameliorated or abolished in leukemic mice receiving RBDS supplementation (Fig 5C).

#### Peripheral Blood Cells Population

**Fig 6:**
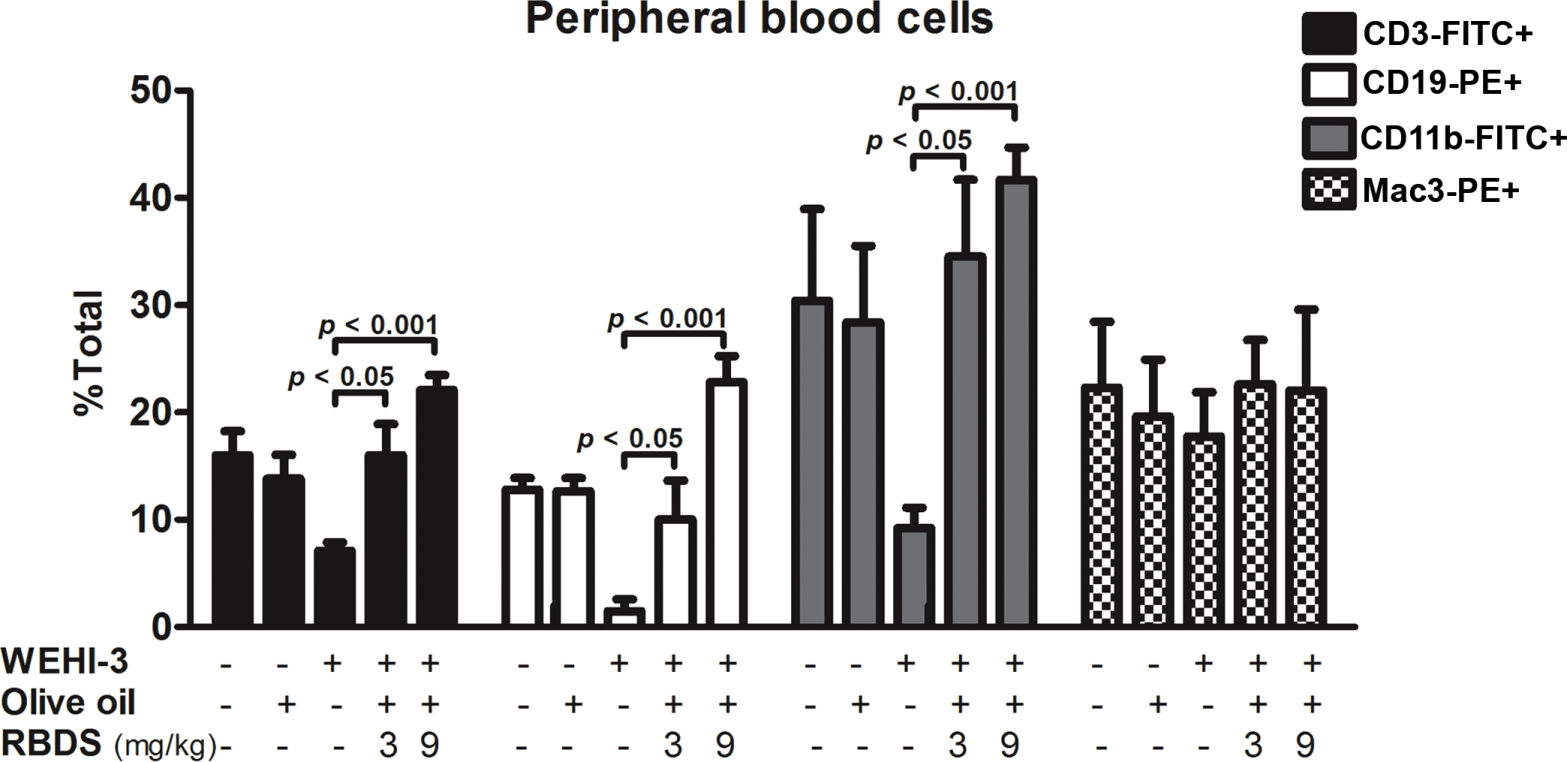
Effect of RBDS on normal immune cells population. The relative amount of normal immune cells in the peripheral blood assessed from the levels of surface markers: CD3^+^ (for T cells), CD19^+^ (for B cells), CD11b^+^ (for monocytes and neutrophils) and Mac3^+^ (for macrophages) in RBDS-treated and untreated leukemic mice. Data are expressed as mean±SEM (n = 10).

Peripheral blood from treated leukemic mice and untreated controls were not conclusively differentiable by routine morphological examination (data not shown). The immunophenotype using flow cytometry analysis for normal (mature) immune cells in the isolates from individual animals of each group demonstrated a significant increase in percentage of CD3^+^ positive (T cells), CD19^+^ positive (B cells), and CD11b^+^ positive (monocytes, neutrophils) in the RBDS-treated leukemic mice (WEHI-3/RBDS 3 mg and WEHI-3/RBDS 9 mg) but not a significant difference of Mac3^+^ positive (macrophage) cells compared to the untreated-WEHI-3 mice (Fig 6).

#### Serum Cytokine Level

**Fig 7:**
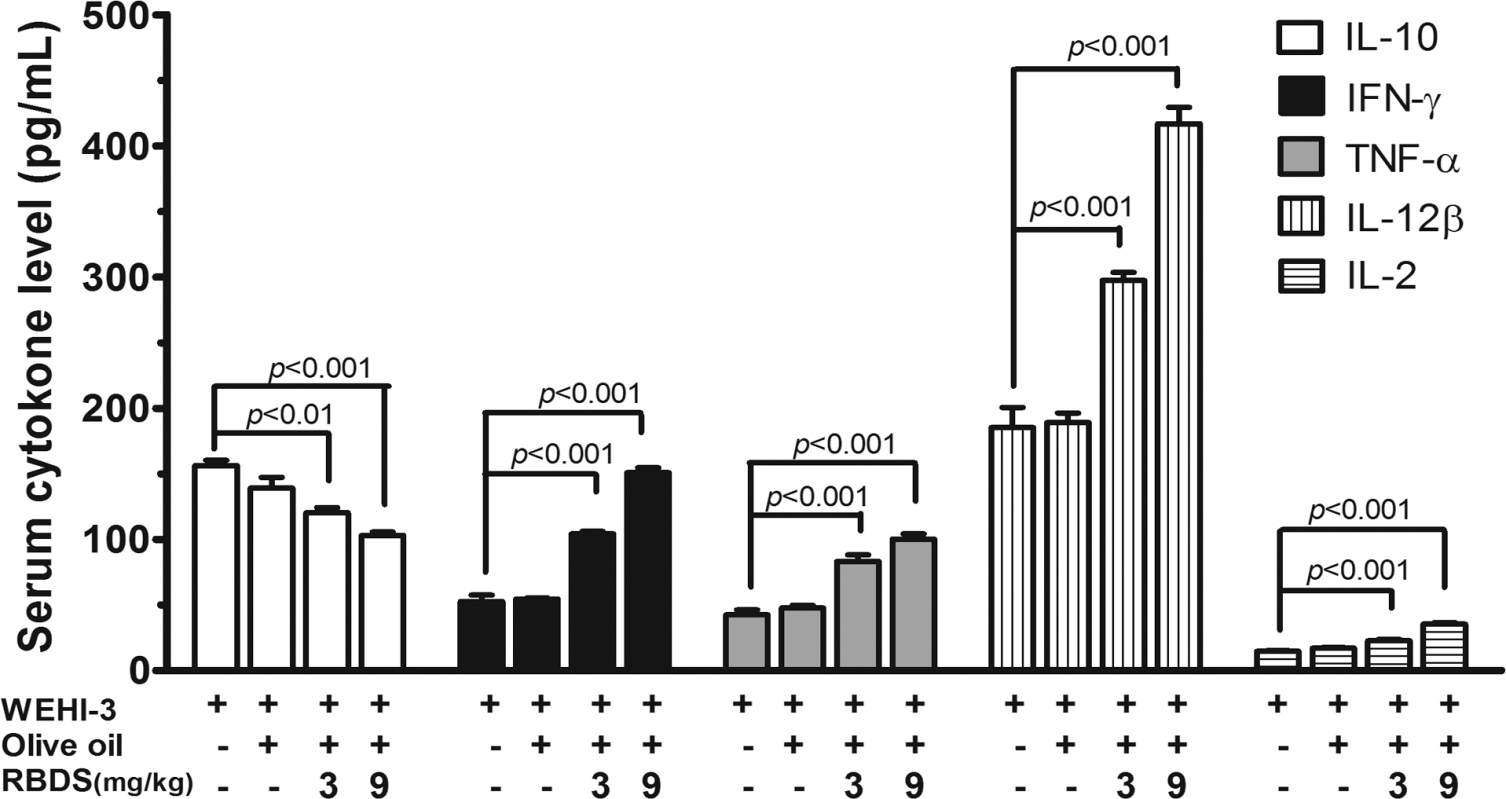
The serum cytokines released from the immune function-related cells of leukemic mice. The induced-leukemic mice were treated with or without RBDS supplementation. Serum cytokines were measured individually from each mouse (four mice per group) by ELISA. Data are expressed as the mean±SEM.

Quantitative measurement of the serum cytokines in mice after the induction of leukemia (WEHI-3) revealed the IL-10 level at 156.30±4.10 pg/mL, whereas the levels of IFN-γ, TNF-α, IL-12β, and IL-2 were 52.63±5.03, 42.63±3.77, 185.40±15.24, and 14.81±0.74 pg/mL, respectively. These cytokines were not significantly changed in the mice receiving olive oil treatment (Fig 7). However, treatment with RBDS (3 mg/kg and 9 mg/kg) significantly decreased the serum IL-10 level, but increased the serum IFN-γ, TNF-α, IL-12β, and IL-2 levels relative to the untreated or olive oil-treated leukemic controls (Fig 7). The change of serum cytokines by the RBDS was exhibited in a concentration dependent manner.

#### Peritoneal Immune Cells and Their Phagocytotic Activity

**Fig 8:**
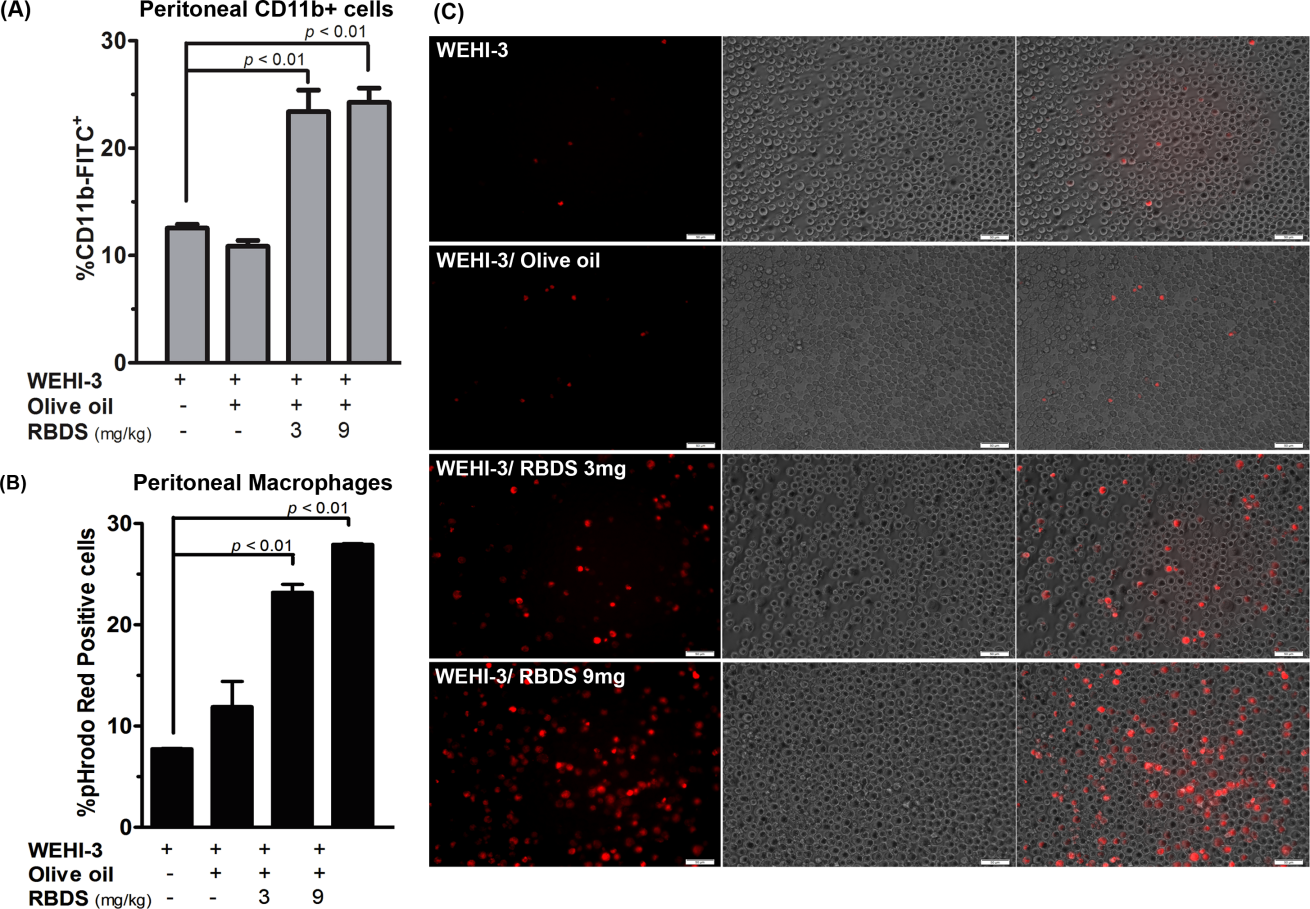
Effect of RBDS containing gramisterol on immune cell population and function. (A) The percentage of peritoneal immune cells assessed from the surface markers level (CD11b^+^) in RBDS-treated and untreated leukemic mice. The experiment was performed in triplicate. Data are expressed as mean±SEM (n = 10). (B) Flow cytometry analysis of peritoneal macrophages phagocytic activity as measured in pHrodo Red positive cells. Data are expressed as mean±SEM (n = 10). (C) Immunofluorescence intensity illustrated by pHrodo Red positive cells represented the phagocytotic activity of the cells.

There was a significant increase in population of CD11b^+^ positive cells in peritoneal lavage from the RBDS-treated leukemic mice (WEHI-3/RBDS 3 mg and WEHI-3/RBDS 9 mg), over the untreated-WEHI-3 and the olive oil-treated controls (Fig 8A). This was well correlated with the increased amount of activated macrophages in the peritoneal lavage. The phagocytotic activity of cells from peritoneal macrophages, as determined from percentage of pHrodo Red incorporated cells using flow cytometry analysis, was significantly increased in leukemic mice treated with RBDS compared to the untreated- and olive oil-treated WEHI-3 mice (Fig 8B). Positive fluorescence cells performing engulfment of pHrodo^TM^ Red-Dextran conjugate were seen in Groups 5–6 (WEHI-3/RBDS 3 mg and WEHI-3/RBDS 9 mg) mice (Fig 8C).

### Signaling Activity of Gramisterol on Normal Immune Cells and Tumor Cells

**Fig 9:**
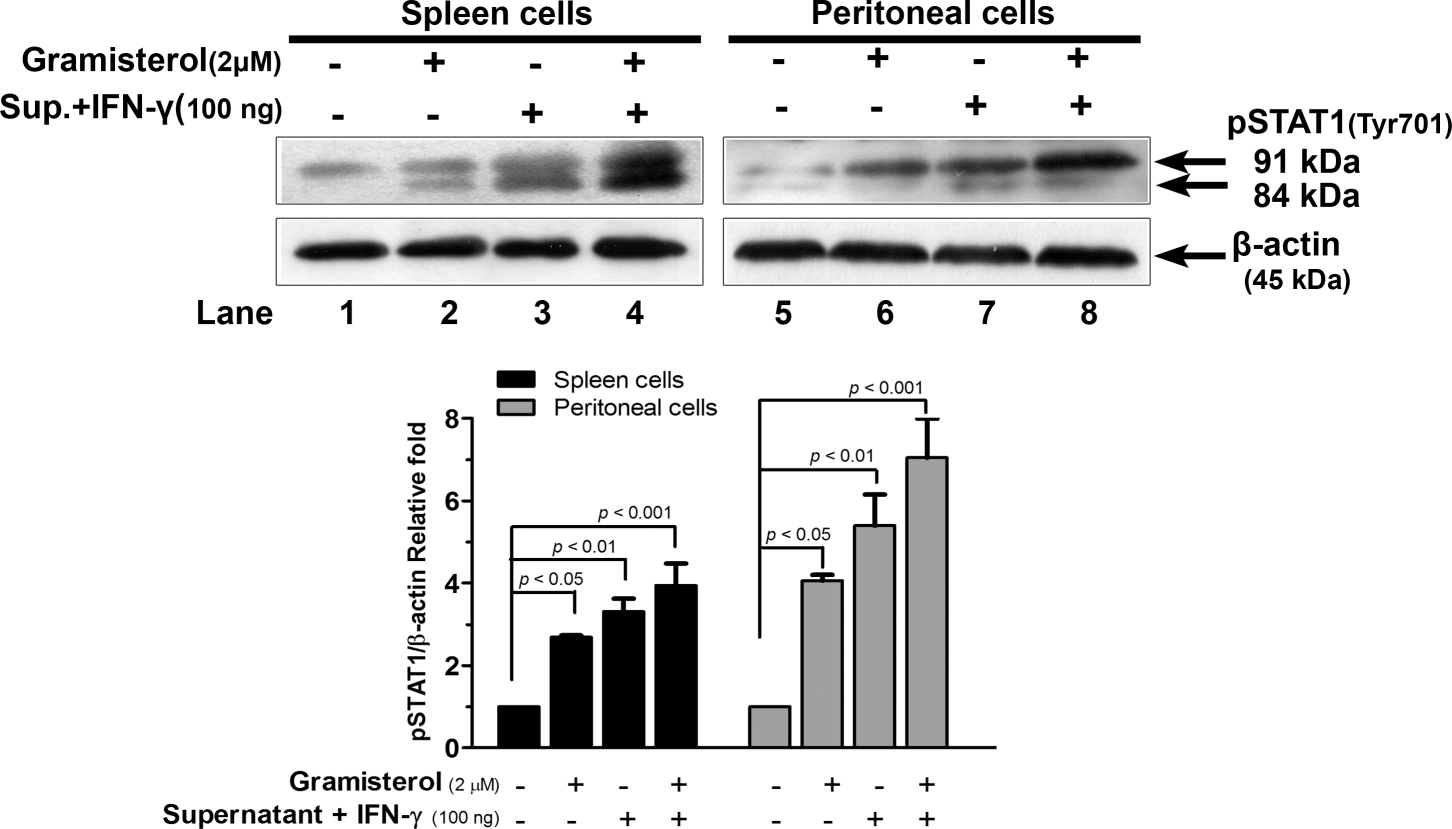
Pathway analysis of gramisterol effect on immune cells. Western blot analysis of pSTAT1 level in spleen cells (lanes 1–4) and peritoneal cells (lanes 5–8) after treatments with i) gramisterol, ii) culture supernatant containing cytokines released from RBDS-treated leukemic mice spleen cells plus a minor supplementation of cytokine IFN-γ, iii) the combination of both gramisterol and the cytokines. β-actin was used as an internal control. The experiment was performed in triplicate. The relative expression of pSTAT1 is shown as mean±SEM.

Treatment of normal immune cells, obtained from normal mice spleen and peritoneal lavage, with gramisterol or the culture supernatant containing cytokines released from RBDS-treated leukemic mice spleen cells plus a minor supplementation of cytokine (100 ng/mL recombinant mouse IFN-γ), increased pSTAT1 signaling in the normal spleen cells and peritoneal cells (Fig 9 lanes 2 and 3 and lanes 6 and 7, respectively). The combination treatment of both gramisterol and the cytokines synergistically enhanced the pSTAT1 signaling in spleen and peritoneal cells (Fig 9 lane 4 and lane 8). In contrast, treatment of WEHI-3 tumor cells with gramisterol or the combination of gramisterol and the cytokines had no significant effect on pSTAT1 activation (data not shown).

### Signaling Mechanism of Gramisterol on WEHI-3 Cells

**Fig 10:**
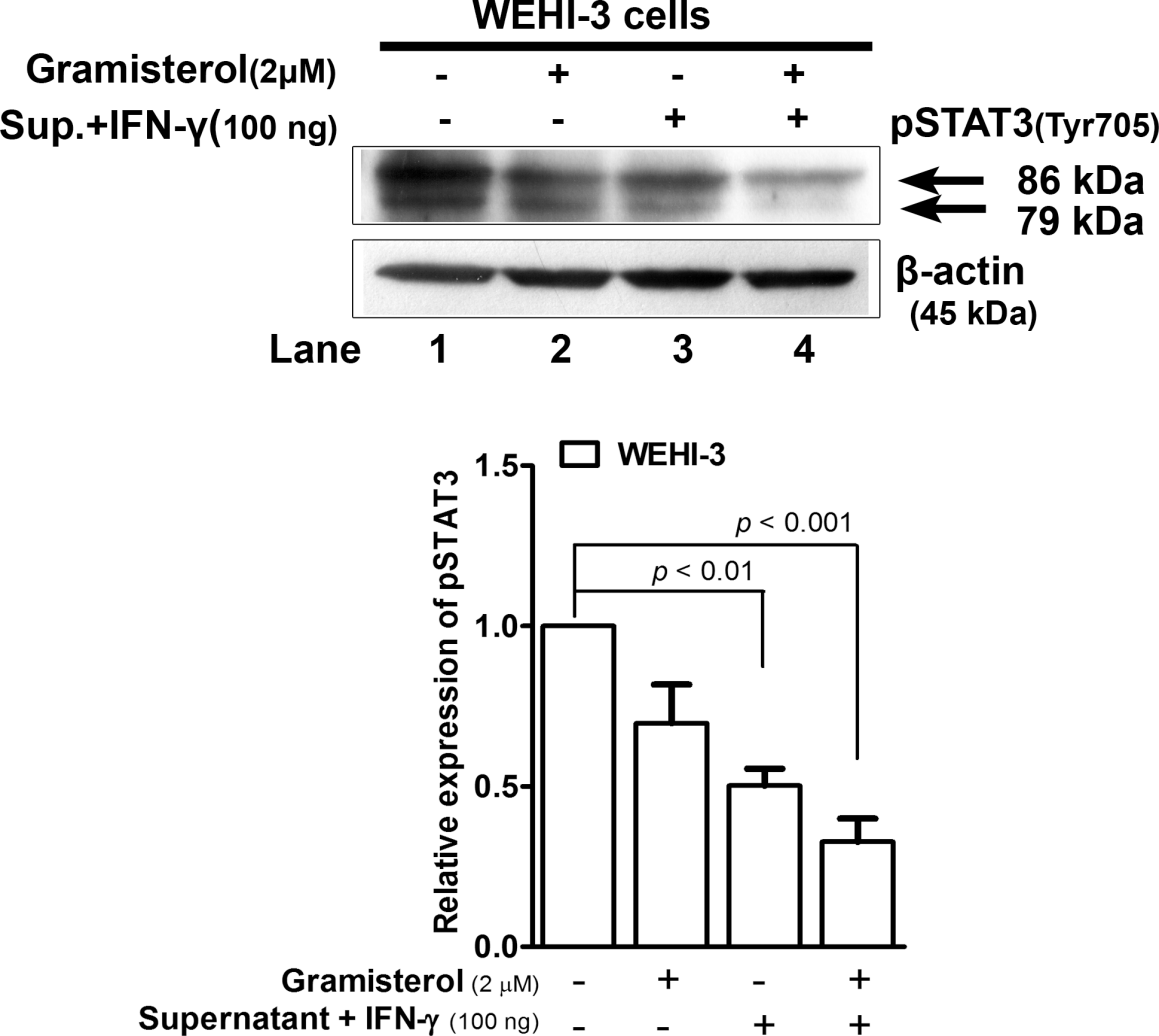
Gramisterol and IFN-γ inhibited STAT3 phosphorylation in tumor cells. Western blot analysis of pSTAT3 signaling activation in WEHI-3 cells after treatment with i) gramisterol, ii) culture supernatant containing cytokines released from RBDS-treated leukemic mice spleen cells plus a minor supplementation of cytokine IFN-γ, iii) the combination of both gramisterol and the cytokines. β-actin was used as an internal control. The experiment was performed in triplicate. The relative expression of pSTAT3 was shown as mean±SEM.

The potential molecular mechanism of gramisterol on the tumor WEHI-3 cells was investigated by treatment of the cells with i) gramisterol, ii) culture supernatant containing cytokines released from RBDS-treated leukemic mice spleen cells plus a minor supplementation of cytokine and iii) the combination of gramisterol and the cytokines. Western blot analysis for activation of STAT3 signaling showed significant decrease of pSTAT3 (Fig 10 lanes 2 and 3) from gramisterol and cytokines treatments alone compared to the untreated group (Fig 10 lane 1). The combination of i) and ii) treatments markedly decreased the pSTAT3 expression level (Fig 10 lane 4).

## Discussion

Gramisterol, the major phytosterol constituent of RBDS, exhibited potent cytotoxic effect against leukemic blood cells. Its powerful *in vitro* effects were manifest by arrest of the sub-G1 phase cell cycle leading to division inhibition and apoptosis progression. Significant decrease of two cell cycle control proteins, cyclin D1 and cyclin E, that regulate the transition of cells from quiescence phase to S phase (G1/S checkpoint) was exhibited in RBDS and gramisterol-treated WEHI-3 cells. The subsequent reduction of DNA accumulation in the S (DNA synthesis) and M (mitosis) phases supports the anti-cell growth and proliferation effects of RBDS and gramisterol [21, 22]. The death effects exerted on WEHI-3 cells by gramisterol or RBDS were mediated via the internal apoptosis pathway in which caspase-3 was activated [23]. In the treated cells, p53 tumor suppressor and Bax pro-apoptosis proteins were subsequently increased in association with a decrease of Bcl-2 anti-apoptosis. These proteins changes during the early apoptosis stage were associated with the changes of mitochondrial membrane and the autocatalytic activation of caspase-3 enzymes (cleaved caspase-3) which eventually signaled the cells to undergo apoptosis [24]. We observed that DNA fragmentation and apoptosis effects in the RBDS-treated cells were concentration-dependent, and when the treatment was prolonged with the higher concentration, the apoptosis (annexin-V staining) could be extended into the late-stage which was an irreversible phenomenon. This cell death inducing effect of the RBDS or its more defined chemical constituent, gramisterol, was consistent with our previous study of the *Riceberry* bran DCM extract in human acute myeloid leukemia cells [17]. In comparison based on the IC_50_, gramisterol exerted a higher efficacy than the RBDS and the DCM extract, respectively.

Administration of RBDS containing gramisterol to mice receiving intraperitoneal transplantation of WEHI-3 cells for a total 28 d remarkably abolished the abnormal leukemia-like conditions, improved the mice immune function and increased the mice survival rate. During the leukemic induction, both innate and adaptive immune responses gradually decreased in mice. Without any treatment, some mice died within 17 d from weakness and distribution of the tumor cells through the blood circulation. At the end, tumor cells accumulated in the spleen and liver. The RBDS-treated leukemic mice had increased numbers of most functional-related immune cells in the peripheral circulation. For example CD^+^3 T cells subpopulation is involved in activation of T cells that participate in innate immune response to eliminate the tumor cells [25]. CD19^+^ B cells function in B cell development which is associated with antibody production [26]. CD11b^+^ leukocytes including monocytes, macrophage, natural killer (NK), neutrophil and B cells are involved in adhesion and leukocytes migration to mediate inflammatory response, and memory B cell migration [27]. In this experiment, the induced leukemic mice expressed high level of serum IL-10 which inhibited phagocytotic activities of macrophages and NK cells leading to innate immune insufficiency [28]. Moreover, IL-10 negatively inhibited the pro-inflammatory cytokines IFN-γ, TNF-α, IL-12β, and IL-2 that play roles in adaptive immune response [29]. Administration of RBDS decreased level of serum IL-10 which allowed myeloid dendritic cells, T-helper cells, and macrophages to achieve tumoricidal capacity [28]. In addition, increase of the pro-inflammatory cytokines was responsible for controlling and linking of the innate and adaptive immunity that affected the tumor cells. For example, high level of TNF-α promoted recruitment of CD11b^+^ cells into the peritoneum causing increase in amount and their phagocytotic activities. TNF-α also promoted MHC antigen expression which enhanced the cognate interactions between antigen-presenting cells and T-cells, thus strengthen the activities of cytotoxic T lymphocytes [30]. Significant increase of IFN-γ (type II IFN) stimulated macrophage and B cell activities, induced T-helper1 cell differentiation [31], and participated in IL-12β and IL-2 production [32, 33]. In this case, IFN-γ together with IL-12β could activate antibody isotype switching that stimulates the complement cascade and opsonization of tumor cells leading to activation of macrophages and enhancing of NK-cells cytotoxic activity [34]. Besides, the increase of IL-2 played a role in activation of naïve helper T lymphocyte proliferation and differentiation to effector T helper cells (T_H_1) [35], which then induced phagocyte activation by triggering macrophages [36]. All of these resulted in immune modulation and improvement of the mice leukemic condition.

Gramisterol exhibited its intracellular targeting toward the normal immune cells as well as the tumor cells. In this animal model, some normal blood cells were expected to remain in lymphoid organs such as the spleen. The splenocytes including hematopoietic cells were eligible for activation with drugs or external supplements. As mentioned above, during the induced AML, production of the cytokines IFN-γ, TNF-α, IL-12β, and IL-2 was inhibited by IL-10 resulting in low STATs signaling activity. Gramisterol mediated the immuno-regulatory process of the leukemic mice by activation of the splenocytes or hematopoietic cells survival and functions via pSTAT1 (signal transducer and activator of transcription 1) signaling. The increased Tyr 701 phosphorylation of STAT1 in gramisterol or cytokines from RBDS-treated splenocytes culture and IFN-γ treated cells suggested that the undifferentiated immune associated cells including T-lymphocytes could be stimulated to survive and perform normal functions. Mechanistically, pSTAT1 was translocated into the nucleus, and acted as a transcriptional control for expression of genes controlling T-cell proliferation, differentiation and migration. In addition, STAT1 could be induced during the proliferative response of T-cell receptor stimulation by IFN-γ [37]. Therefore, combination of gramisterol and IFN-γ treatment could enhance splenocytes activity in fighting the immune deficient condition in AML. Besides, gramisterol targeted WEHI-3 tumor cells by induction of apoptosis via interruption of an oncogenic transcription factor, pSTAT3 which was overexpressed in primary human tumors included AML as well as the WEHI-3 cells [38]. The decreased Tyr 705 phosphorylation of STAT3 in gramisterol or cytokines from RBDS-treated splenocytes culture and IFN-γ treated cells resulted in transcription inhibition of the genes for growth and proliferation of the tumor cells. Thus, combination of gramisterol and IFN-γ treatment could eliminate the tumor cells in AML.

Although STATs could be activated by many factors including carcinogens, growth factors, oncogenes, and inflammatory cytokines [39], we found that different STATs were activated in cells after treatment with gramisterol. Generally, the binding of IL-10 to the receptor on immune cells activates tyrosine kinase-2 (TYK-2) and induces phosphorylation of its downstream molecule (STAT3) to increase Bcl-2 expression resulting in cell survival and functions [40]. However, when WEHI-3 cells received gramisterol with or without cytokines from the splenocytes culture, phosphorylation of STAT3 was decreased. This evidence strongly suggests that the inhibitory effect of gramisterol on Bcl-2 induced WEHI-3 survival was an indirect effect mediated through pSTAT3 signaling. Gramisterol exerted direct inhibition on or disruption of pSTAT3 transcriptional controlling of WEHI-3 cell survival genes including Bcl-2 expression. This assumption supports the previous study demonstrating that the down-regulation of Bcl-2 can be a result of STAT3 inhibitory effect [41]. The combined inhibitory effect of gramisterol and IFN-γ against pSTAT3 was significant in a dose-dependent manner leading to apoptosis of the tumor cells.

Taken together, the data suggested the potential medicinal application of rice bran gramisterol as a target anti-cancer drug against AML. This natural health compound is an ingredient found in the RBDS (yield = 56.6%) which can be DCM extracted from the bran of *Riceberry* rice [17]. Gramisterol possesses a potent pSTAT3 signaling inhibition leading to tumor cell death, but enhances pSTAT1 signaling that mediates hematopoietic cell survival and modulates the immune functions of T-cells in AML.

## References

[1] Kaufman DW, Anderson TE, Issaragrisil S. Risk factors for leukemia in Thailand. Ann Hematol. 2009;88(11):1079–88. 10.1007/s00277-009-0731-9 19294385

[2] Hayes RB, Songnian Y, Dosemeci M, Linet M. Benzene and lymphohematopoietic malignancies in humans. Am J Ind Med. 2001;40(2):117–26. 11494338 10.1002/ajim.1078

[3] Savitz DA, Andrews KW. Review of epidemiologic evidence on benzene and lymphatic and hematopoietic cancers. Am J Ind Med. 1997;31(3):287–95. 9055951 10.1002/(sici)1097-0274(199703)31:3<287::aid-ajim4>3.0.co;2-v

[4] Pearce MS, Salotti JA, Little MP, McHugh K, Lee C, Kim KP, et al Radiation exposure from CT scans in childhood and subsequent risk of leukaemia and brain tumours: a retrospective cohort study. Lancet. 2012;380(9840):499–505. 10.1016/S0140-6736(12)60815-0 22681860 PMC3418594

[5] Linet MS, Kim KP, Rajaraman P. Children’s exposure to diagnostic medical radiation and cancer risk: epidemiologic and dosimetric considerations. PediatrRadiol. 2009;39(1):4–26. 10.1007/s00247-008-1026-3 PMC2814780 19083224

[6] Ron E. Ionizing radiation and cancer risk: evidence from epidemiology. Radiat Res. 1998;150(5s):S30–S41. 9806607

[7] Travis LB, Holowaty EJ, Bergfeldt K, Lynch CF, Kohler BA, Wiklund T, et al Risk of leukemia after platinum-based chemotherapy for ovarian cancer. N Engl J Med. 1999;340(5):351–7. 9929525 10.1056/NEJM199902043400504

[8] Curtis RE, Boice JD Jr, Stovall M, Bernstein L, Greenberg RS, Flannery JT, et al Risk of leukemia after chemotherapy and radiation treatment for breast cancer. N Engl J Med. 1992;326(26):1745–51. 1594016 10.1056/NEJM199206253262605

[9] DiNardo CD, Cortes JE. New treatment for acute myelogenous leukemia. Expert OpinPharmacother. 2015;16(1):95–106. 10.1517/14656566.2015.981527 25480777

[10] Fernandez HF, Sun Z, Yao X, Litzow MR, Luger SM, Paietta EM, et al Anthracycline dose intensification in acute myeloid leukemia. N Engl J Med. 2009;361(13):1249–59. 10.1056/NEJMoa0904544 19776406 PMC4480917

[11] Schechter T, Gassas A, Chen H, Pollard J, Meshinchi S, Zaidman I, et al The outcome of allogeneic hematopoietic cell transplantation for children with FMS-like tyrosine kinase 3 internal tandem duplication-positive acute myelogenous leukemia. BiolBloodMarrowTransplant. 2015;21(1):172–5. 10.1016/j.bbmt.2014.08.008 25139215

[12] Aguilar-Garcia C, Gavino G, Baragaño-Mosqueda M, Hevia P, Gavino VC. Correlation of tocopherol, tocotrienol, γ-oryzanol and total polyphenol content in rice bran with different antioxidant capacity assays. Food Chem. 2007;102(4): 1228–32.

[13] Ardiansyah, Shirakawa H, Koseki T, Ohinata K, Hashizume K, Komai M. Rice bran fractions improve blood pressure, lipid profile, and glucose metabolism in stroke-prone spontaneously hypertensive rats. J Agric Food Chem. 2006;54(5):1914–20. 16506853 10.1021/jf052561l

[14] Jariwalla R. Rice-bran products: phytonutrients with potential applications in preventive and clinical medicine. Drugs ExpClin Res. 2000;27(1):17–26. 11276826

[15] Min B, McClung AM, Chen MH. Phytochemicals and antioxidant capacities in rice brans of different color. J Food Sci. 2011;76(1):C117–C26. 10.1111/j.1750-3841.2010.01929.x 21535639

[16] Sriseadka T, Wongpornchai S, Rayanakorn M. Quantification of flavonoids in black rice by liquid chromatography-negative electrospray ionization tandem mass spectrometry. J Agric Food Chem. 2012;60(47):11723–32. 10.1021/jf303204s 23121250

[17] Leardkamolkarn V, Thongthep W, Suttiarporn P, Kongkachuichai R, Wongpornchai S, Wanavijitr A. Chemopreventive properties of the bran extracted from a newly-developed Thai rice: The *Riceberry*. Food Chem. 2011;125(3):978–85.

[18] Prangthip P, Surasiang R, Charoensiri R, Leardkamolkarn V, Komindr S, Yamborisut U, et al Amelioration of hyperglycemia, hyperlipidemia, oxidative stress and inflammation in steptozotocin-induced diabetic rats fed a high fat diet by riceberry supplement. J Funct Foods. 2013;5(1): 195–203.

[19] Suttiarporn P, Chumpolsri W, Mahatheeranont S, Luangkamin S, Teepsawang S, Leardkamolkarn V. Structures of phytosterols and triterpenoids with potential anti-cancer activity in bran of black non-glutinous rice. Nutrients. 2015;7(3): 1672–87. 10.3390/nu7031672 25756784 PMC4377873

[20] Warner NL, Moore MA, Metcalf D. A transplantable myelomonocytic leukemia in BALB/c mice: cytology, karyotype, and muramidase content. J Natl Cancer Inst. 1969;43(4):963–82. 5259325

[21] Mertens-Talcott SU, Percival SS. Ellagic acid and quercetin interact synergistically with resveratrol in the induction of apoptosis and cause transient cell cycle arrest in human leukemia cells. Cancer Lett. 2005;218(2):141–51. 15670891 10.1016/j.canlet.2004.06.007

[22] Hartwell LH, Kastan MB. Cell cycle control and cancer. Science. 1994;266(5192):1821–8. 7997877 10.1126/science.7997877

[23] Lin H-I, Lee Y-J, Chen B-F, Tsai M-C, Lu J-L, Chou C-J, et al Involvement of Bcl-2 family, cytochrome and caspase 3 in induction of apoptosis by beauvericin in human non-small cell lung cancer cells. Cancer Lett. 2005;230(2):248–59. 16297711 10.1016/j.canlet.2004.12.044

[24] Jin Z, El-Deiry WS. Review overview of cell death signaling pathways. Cancer BiolTher. 2005;4(2):139–63. 10.4161/cbt.4.2.1508 15725726

[25] Ishigami S, Natsugoe S, Tokuda K, Nakajo A, Xiangming C, Iwashige H, et al Clinical impact of intratumoral natural killer cell and dendritic cell infiltration in gastric cancer. Cancer Lett. 2000;159(1):103–8. 10974412 10.1016/s0304-3835(00)00542-5

[26] Hansen MH, Nielsen H, Ditzel HJ. The tumor-infiltrating B cell response in medullary breast cancer is oligoclonal and directed against the autoantigen actin exposed on the surface of apoptotic cancer cells. Proc Natl Acad Sci. 2001;98(22):12659–64. 11606714 10.1073/pnas.171460798 PMC60110

[27] Han C, Jin J, Xu S, Liu H, Li N, Cao X. Integrin CD11b negatively regulates TLR-triggered inflammatory responses by activating Syk and promoting degradation of MyD88 and TRIF via Cbl-b. Nat Immunol. 2010;11(8):734–42. 10.1038/ni.1908 20639876

[28] Saraiva M, O'Garra A. The regulation of IL-10 production by immune cells. Nat Rev Immunol. 2010;10(3):170–81. 10.1038/nri2711 20154735

[29] Ding Y, Chen D, Tarcsafalvi A, Su R, Qin L, Bromberg JS. Suppressor of cytokine signaling 1 inhibits IL-10-mediated immune responses. J Immunol. 2003;170(3):1383–91. 12538698 10.4049/jimmunol.170.3.1383

[30] Zhang W, Chen Z, Li F, Kamencic H, Juurlink B, Gordon JR, et al Tumour necrosis factor‐α (TNF‐α) transgene‐expressing dendritic cells (DCs) undergo augmented cellular maturation and induce more robust T‐cell activation and anti‐tumour immunity than DCs generated in recombinant TNF‐α. Immunol. 2003;108(2):177–88. 10.1046/j.1365-2567.2003.01489.x PMC1782887 12562326

[31] Schroder K, Hertzog PJ, Ravasi T, Hume DA. Interferon-γ: an overview of signals, mechanisms and functions. J Leukoc Biol. 2004;75(2):163–89. 14525967 10.1189/jlb.0603252

[32] Trinchieri G. Interleukin-12: a cytokine produced by antigen-presenting cells with immunoregulatory functions in the generation of T-helper cells type 1 and cytotoxic lymphocytes. Blood. 1994;84(12): 4008–27. 7994020

[33] Brown TJ, Lioubin MN, Marquardt H. Purification and characterization of cytostatic lymphokines produced by activated human T lymphocytes. Synergistic antiproliferative activity of transforming growth factor beta 1, interferon-gamma, and oncostatin M for human melanoma cells. J Immunol. 1987;139(9): 2977–83. 3117884

[34] Colombo MP, Trinchieri G. Interleukin-12 in anti-tumor immunity and immunotherapy. Cytokine Growth Factor Rev. 2002;13(2): 155–68. 11900991 10.1016/s1359-6101(01)00032-6

[35] Boyman O, Sprent J. The role of interleukin-2 during homeostasis and activation of the immune system. Nat Rev Immunol. 2012;12(3): 180–90. 10.1038/nri3156 22343569

[36] Wang SY, Hsu ML, Hsu HC, Lee SS, Shiao MS, Ho CK. The anti‐tumor effect of *Ganoderma lucidum* is mediated by cytokines released from activated macrophages and T lymphocytes. Int J Cancer. 1997;70(6): 699–705. 9096652 10.1002/(sici)1097-0215(19970317)70:6<699::aid-ijc12>3.0.co;2-5

[37] Lee C-K, Smith E, Gimeno R, Gertner R, Levy DE. STAT1 affects lymphocyte survival and proliferation partially independent of its role downstream of IFN-γ. J Immunol. 2000;164(3): 1286–92. 10640742 10.4049/jimmunol.164.3.1286

[38] Gouilleux-Gruart V, Gouilleux F, Desaint C, Claisse J, Capiod J-C, Delobel J, et al STAT-related transcription factors are constitutively activated in peripheral blood cells from acute leukemia patients. Blood. 1996;87(5): 1692–7. 8634413

[39] Yu H, Pardoll D, Jove R. STATs in cancer inflammation and immunity: a leading role for STAT3. Nat Rev Cancer. 2009;9(11): 798–809. 10.1038/nrc2734 19851315 PMC4856025

[40] Weber-Nordt R, Henschler R, Schott E, Wehinger J, Behringer D, Mertelsmann R, et al Interleukin-10 increases Bcl-2 expression and survival in primary. Blood. 1996;88(7): 2549–58. 8839847

[41] Pensa S, Regis G, Boselli D, Novelli F, Poli V. STAT1 and STAT3 in tumorigenesis: two sides of the same coin? In: Stephanou A., editor. JAK-Stat Pathway in Disease. Austin: Landes Bioscience; 2000pp. 100–121.

